# Differential-Privacy-Based Collaborative Protection for Visual and Location Data in UAV Semantic Communications

**DOI:** 10.3390/s26144358

**Published:** 2026-07-09

**Authors:** Sitang Yue, Chong Zhan, Guanwu Jiang, Yingmin Qiu, Shujun Han

**Affiliations:** 1 School of Cyberspace Security, Beijing University of Posts and Telecommunications, Beijing 100876, China; sitangyue@bupt.edu.cn (S.Y.); zhanchong@bupt.edu.cn (C.Z.); ymq@bupt.edu.cn (Y.Q.); 2 School of Information and Communication Engineering, Beijing University of Posts and Telecommunications, Beijing 100876, China; jiangguanwu@bupt.edu.cn

**Keywords:** UAV, semantic communication, differential privacy, visual information privacy-preserving, location information privacy-preserving, joint transmission power and privacy budget optimization

## Abstract

Unmanned aerial vehicle semantic communications are increasingly required in low-altitude sensing, intelligent inspection, and emergency response, where raw image transmission is difficult to sustain under limited onboard resources and time-varying air-to-ground links. Meanwhile, the simultaneous transmission of visual semantic features and object-centre location metadata under third-party eavesdropping creates a dual-privacy vulnerability: an attacker can exploit both to reconstruct sensitive content. In this paper, we propose a differential privacy-based collaborative protection framework that inserts dedicated perturbations into visual semantic and location descriptors before transmission. For visual data, we design a region-aware differential privacy mechanism that applies stronger noise to sensitive semantic regions while preserving utility for non-critical areas. For location data, a scenario-adaptive strategy is developed, comprising randomized differential privacy for discrete grid-based location information (coarse spatial awareness) and Laplace-based differential privacy for continuous coordinates (fine-grained protection). To balance privacy and utility, we formulate a joint optimization problem. It maximizes legitimate-side semantic task performance by coordinating the visual privacy budget, location privacy budget, and transmit power. A BCD-based algorithm is developed to solve this non-convex problem. Attacker-side recoverability is verified empirically at the optimized operating point. Simulation results demonstrate stable convergence within a small number of iterations. Compared with uniform differential privacy, the proposed framework achieves a superior task-level privacy–utility trade-off and provides selective sensitive-region protection, with the two mechanisms yielding comparable whole-image attack suppression.

## 1. Introduction

Unmanned aerial vehicles (UAVs) are increasingly used in low-altitude sensing, intelligent inspection, and emergency response. In these applications, the UAV continuously captures visual scenes and reports task-relevant information to the ground side. However, raw image transmission is difficult to sustain under the bandwidth, transmit-power, and onboard computing constraints of UAV platforms. Semantic communication alleviates this bottleneck by shifting the focus from bit-level fidelity to task-oriented meaning. Recent surveys and reviews have further clarified its theoretical basis, task-oriented objective, and future-Internet relevance in emerging wireless and AI-native systems [1,2,3]. Recent implementation-oriented discussions also address semantic transmission compatibility with digital wireless systems and cloud-edge-device collaborative settings [4,5]. For image-oriented transmission, recent semantic coding studies also show that useful task semantics can be preserved under constrained bandwidth and dynamic channels through task-aware representation learning and lightweight semantic reconstruction [6,7]. UAV-based visual sensing is an important application domain for such systems: robust aerial semantic segmentation under resource constraints has been studied in [8], and edge perception frameworks for next-generation networks with semantic awareness are discussed in [9]. Recent high-quality studies further investigate distortion-resilient goal-oriented transmission and privacy-aware image JSCC [10,11], while recent implementations continue to explore adaptive image transmission under resource constraints [12].

However, in UAV semantic communication, the key challenge is no longer efficient transmission alone, but privacy-aware control under coupled resource constraints. On the visual side, intermediate semantic features may still preserve contours, scene structure, and category cues, leaving the system vulnerable to semantic inference, feature analysis, model inversion, or model-theft-assisted eavesdropping [13,14,15]. Broader security reviews and recent secure semantic transmission studies likewise emphasize adaptive and context-aware protection as a growing theme in semantic secure communications [15,16]. On the spatial side, bounding-box centers and derived location descriptors can reveal target positions, mission areas, and mobility traces, which remain high-risk structured spatial data in recent edge offloading privacy, trajectory privacy publishing, and privacy-preserving UAV location-authentication studies [17,18,19]. UAV-specific collaborative sensing studies further show that distributed localization and fusion can also leak geometric state information unless privacy-preserving masking is incorporated into the fusion stage [20]. Recent mobility-oriented semantic communication surveys likewise emphasize that semantic messages may leak trajectories and contextual behavior unless privacy and security are designed together [21]. Related UAV privacy studies also show that privacy-preserving offloading and geofence-aware authentication are becoming practical requirements for resource-constrained aerial systems [19,22]. Meanwhile, UAV platforms must operate under limited transmit power, fluctuating air–ground channels, and constrained onboard computation. Therefore, the core problem addressed in this paper is how to jointly suppress visual and location privacy leakage while preserving legitimate task recovery in a resource-constrained UAV semantic communication link.

Existing studies remain insufficient in four closely related respects. First, semantic communication foundations remain task-oriented rather than dual-privacy-coupled. Recent reviews and representative studies consolidate the semantic communication paradigm and task-oriented transmission principles [1,2,6]. However, they do not address coordinated visual and location privacy in UAV sensing scenarios. Second, semantic transmission adaptation and edge-inference optimization still emphasize utility maximization rather than joint privacy control. Existing studies improve task utility and resource efficiency under dynamic constraints [4,23]. However, they do not coordinate visual privacy, location privacy, and transmit-power budgets within one framework. Third, secure semantic communication studies mainly focus on visual leakage, model security, or representation inversion. They clarify semantic-feature privacy risks and the need for representation-level protection [11,13,14]. However, they usually omit location leakage carried by object descriptors. Fourth, location privacy studies provide useful spatial protection tools, but rarely integrate them into semantic communication utility optimization. Edge offloading privacy, trajectory publishing, and UAV location authentication address important facets of spatial disclosure [17,18,19]. UAV-specific distributed localization privacy is also emerging [20]. However, these strands are seldom coupled with semantic decoding quality and air–ground resource allocation in a unified UAV setting. Therefore, a unified setting that controls legitimate-receiver task utility, attacker-side recoverability, and communication-resource budgets is still lacking.

Accordingly, this paper develops a privacy-enhanced UAV semantic communication framework in which dedicated perturbation mechanisms are inserted into both the visual semantic stream and the location-descriptor stream before transmission. The main contributions are summarized as follows:

1. **Dual-Privacy UAV Semantic Communication Framework:** We establish a unified system model that couples dual-privacy modeling—visual semantic leakage and location-descriptor leakage—with communication-resource constraints in a single UAV air–ground link. Unlike prior work that either protects only visual features [11,13] or handles location privacy in isolation [17,18], the proposed framework explicitly coordinates both protection streams and their interaction with transmit power, establishing a principled privacy–utility operating point for the legitimate receiver while suppressing attacker-side recoverability.
2. **Differential-Privacy-based Visual and Location Protection Mechanisms:** For visual data, we design a region-aware DP mechanism that constructs a class-conditioned, cell-wise privacy-budget map from the global budget $\epsilon_{v}$, applying stronger Gaussian noise to sensitive semantic regions (e.g., pedestrians, vehicles) while preserving utility in non-critical areas. The semantic grounding of the perturbation map—derived from task-level object detections and the feature-grid coordinate transform—directly links the protection intensity to the task-oriented nature of the semantic representation. For location data, we propose a scenario-adaptive strategy that selects between randomized DP on a discrete grid and planar Laplace geo-indistinguishability according to the spatial granularity required by the mission, both with formal DP guarantees.
3. **Joint Optimization of Privacy Budgets and Transmit Power:** We formulate a scenario-dependent joint utility maximization over the visual privacy budget $\epsilon_{v}$, location privacy budget $\epsilon_{l}$, and transmit power $P_{\mathrm{tx}}$, capturing the coupled influence of privacy noise, channel quality, and resource constraints on both the legitimate receiver’s task performance and the attacker’s recoverability. A BCD-based algorithm is developed to solve this non-convex problem, converging within 6–8 iterations in all tested scenarios.
4. **Experimental Validation:** Simulation results on the VisDrone dataset demonstrate stable convergence, differentiated scenario-adaptive behavior, and a superior privacy–utility trade-off relative to uniform DP. In the fixed-budget surveillance setting, region-aware DP improves semantic class accuracy by 7.1%, reduces sensitive-region SSIM by 60.3%, and lowers sensitive-feature similarity by 18.2% compared with uniform DP, while maintaining comparable whole-image attack suppression.

The rest of this paper is organized as follows. Section 2 presents the UAV semantic communication system model, the receiver/eavesdropper observation boundaries, and the privacy-enhanced transmission model. Section 3 describes the differential-privacy-based visual and location protection mechanisms together with their theoretical guarantees. Section 4 formulates the joint optimization problem of transmit power and privacy budgets and presents the proposed algorithm. Section 5 gives the simulation results and analysis. Section 6 concludes the paper. Table 1 summarises the principal symbols used in this paper.

## 2. System Model

### 2.1. UAV Semantic Communications Under Third-Party Eavesdropping

**Figure 1:**
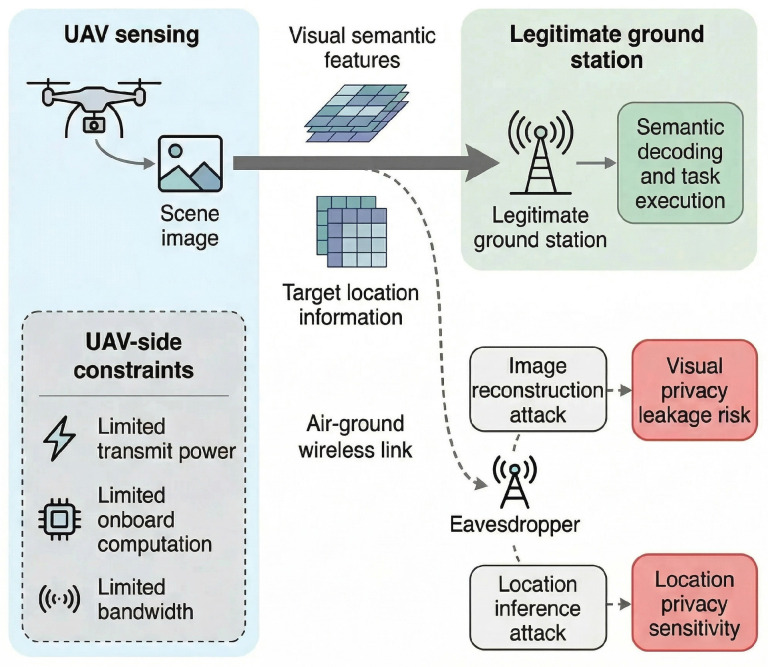
Illustration of the UAV semantic communication system with a third-party eavesdropper, privacy leakage risks, and resource constraints.

Following the encoder–channel–decoder narrative widely adopted in semantic image transmission studies, such as [6], we consider a UAV-assisted air–ground semantic communication system composed of a UAV transmitter, a legitimate ground station, and a third-party passive eavesdropper. Figure 1 illustrates the overall scene, including the UAV sensing side, the legitimate receiver, the passive eavesdropper, and the coupled pressures of privacy leakage and resource constraints.

The UAV semantic communication system with a third-party eavesdropper comprises four stages: transmitter-side semantic encoding, air-to-ground transmission, legitimate receiver decoding, and eavesdropper observation. Specifically, the UAV captures scene images and converts them into task-oriented semantic messages before transmission. The legitimate ground station receives the protected semantic messages and reconstructs a task-oriented output. The passive eavesdropper can intercept the same transmitted variables, but does not belong to the authorized processing chain.

### 2.2. Transmitter-Side Semantic Encoding Model

Let $I\in R^{H_{in}\times W_{in}\times 3}$ denote the input UAV image. The transmitter-side semantic encoder and detector are modelled by

(1) (V,O)=E(I),

where $V\in R^{C\times H^{\prime }\times W^{\prime }}$ is the visual semantic feature tensor and $O=\{O_{1},\ldots ,O_{M}\}$ is the detected object set.

For each detected object $O_{j}$, the transmitter records its bounding box $B_{j}=\left(x_{j},y_{j},w_{j},h_{j}\right)$ and category label $l_{j}\in \left\{1,\ldots ,K\right\}$, where $\left(x_{j},y_{j}\right)$ is the normalized object center and $\left(w_{j},h_{j}\right)$ are the normalized box width and height. The target-related descriptor organizer G(·) is

(2) $$D_{\mathrm{obj}}=G\left(O\right)={\left\{\left(c_{j},w_{j},h_{j},l_{j}\right)\right\}}_{j=1}^{M},$$

where $c_{j}=\left(x_{j},y_{j}\right)\in {\left[0,1\right]}^{2}$ denotes the normalized center coordinate of object *j*.

The tensor V is the main carrier of visual task semantics, whereas $D_{\mathrm{obj}}$ preserves coarse object layout and category metadata. The latter is not introduced as an independent task head; rather, it is a structured semantic description accompanying the visual tensor.

### 2.3. Air–Ground Transmission Model for Visual and Location Messages

At the transmission stage, the UAV places two coupled semantic messages on the air–ground link: a visual semantic tensor and a compact target-related descriptor set. To keep the channel model independent of the concrete protection realization, these transmitted messages are denoted by $V_{\mathrm{tx}}$ and $D_{\mathrm{tx}}$, respectively.

To capture the multipath propagation characteristics of realistic UAV air–ground links, the wireless channel is modelled as a Rayleigh block-fading channel with LoS-dominated large-scale path loss. The effective channel power gain comprises a distance-dependent path-loss term $h_{\mathrm{pl}}$ and a Rayleigh-distributed fading coefficient $h_{f}$ with $|h_{f}{|}^{2}∼\mathrm{Exp}\left(1\right)$ (unit mean), so that $h=h_{\mathrm{pl}}\cdot h_{f}$. The receiver-side thermal noise is represented by additive white Gaussian noise with variance $\sigma_{\mathrm{ch}}^{2}$. Under this model, the transmitted visual tensor and descriptor set are jointly written as

(3) $$\left(\hat{V},{\hat{D}}_{\mathrm{obj}}\right)=C\left(V_{\mathrm{tx}},D_{\mathrm{tx}};h,P_{\mathrm{tx}}\right),$$

where $\hat{V}$ and ${\hat{D}}_{\mathrm{obj}}$ denote the received visual and descriptor messages after air–ground transmission.

Within one transmission block, *h* is assumed to remain constant across blocks. It varies with the UAV-ground geometry and propagation environment. The received SNR at the legitimate receiver is therefore

(4) $$\gamma =\frac{P_{\mathrm{tx}}h}{\sigma_{\mathrm{ch}}^{2}}.$$

The same block-level channel state is used for both message components at the system-model level, so the coupled influence of $P_{\mathrm{tx}}$, *h*, and $\sigma_{\mathrm{ch}}^{2}$ on semantic recovery is summarized through γ for the later analysis.

### 2.4. Legitimate Receiver Semantic Decoding Model

At the legitimate ground station, the received visual semantic tensor $\hat{V}$ is decoded together with detector-derived prior cues reconstructed from the received descriptor set ${\hat{D}}_{\mathrm{obj}}$. Let $P_{\det}$ denote the reconstructed prior cues. These cues encode category-presence information and coarse spatial supports derived from the received object descriptors.

The receiver-side semantic recovery process is written as

(5) $$\hat{M}=D_{\mathrm{sem}}\left(\hat{V};P_{\det}\right),$$

where $D_{\mathrm{sem}}\left(\cdot \right)$ denotes the legitimate-side semantic decoder and $\hat{M}$ is the recovered semantic mask or task-oriented output.

At the receiver, the visual tensor $\hat{V}$ enters the semantic decoder as the main information carrier. The received descriptor set ${\hat{D}}_{\mathrm{obj}}$ is reorganized into $P_{\det}$ rather than passed through a separate location decoder, and then serves as a refinement prior indicating which object categories are present and where coarse object regions are likely to appear.

### 2.5. Threat Model and Observation at the Eavesdropper

In this paper, we consider two coupled privacy risks. The first is visual privacy leakage, in which the intercepted semantic feature tensor still reveals sensitive scene content. The second is location privacy leakage, in which intercepted object descriptors reveal target positions or coarse mission geometry. If either side remains weakly protected, the attacker can fuse the two information sources and improve its recovery ability.

The passive eavesdropper can intercept the same transmitted variables as the legitimate receiver, but it cannot access raw onboard images, modify the transmission protocol, or obtain authorized side information [13,15]. To conservatively model the attacker, we assume that it knows the overall system architecture (a white-box/grey-box assumption) and may employ representation inversion, semantic inference, or joint descriptor-assisted recovery. This paper employs two attacker formulations that probe different capability levels. The gradient-based optimization attacker iteratively minimizes the reconstruction distance between a candidate image and the intercepted protected feature at inference time, without any prior training on the data distribution [13], thereby providing a data-free lower bound on attacker capability. The trained inversion network pre-trains a lightweight convolutional inverter $I:R^{C\times H^{\prime }\times W^{\prime }}\;\to R^{3\times H\times W}$ on in-distribution training images and applies it at inference time to reconstruct the original visual content from the intercepted feature map, without relying on iterative gradient optimisation [14]; as a strictly stronger adversary exploiting in-distribution training data, it validates that the protection conclusions hold under a more capable, realistic threat. Both attackers share the same knowledge boundary: they know the encoder architecture and feature dimensionality but cannot access raw UAV images, DP noise realizations, or authorized side information. The privacy model is defined at the semantic-representation level rather than through physical-layer secrecy assumptions.

At the representation level, the attacker-side visual recovery attempt is abstracted as

(6) $${\hat{I}}_{\mathrm{adv}}=A\left(\hat{V},{\hat{D}}_{\mathrm{obj}}\right),$$

where A(·) denotes a generic inference or attack operator. In addition to visual inversion, the eavesdropper may directly exploit ${\hat{D}}_{\mathrm{obj}}$ or its derived spatial statistics to infer sensitive target positions, activity trajectories, or mission areas.

#### Privacy-Enhanced Semantic Information Transmission

Before entering the air–ground link, the transmitted visual tensor and target-related descriptor set can be privacy-enhanced at the UAV side. In this paper, the visual protection intensity is controlled by $\epsilon_{v}$, the location protection intensity is controlled by $\epsilon_{l}$, and the communication quality is controlled by $P_{\mathrm{tx}}$. At the system level, the transmitted pair is written as

(7) $$V_{\mathrm{tx}}=M_{v}\left(V;\epsilon_{v}\right),\;D_{\mathrm{tx}}=M_{l}\left(D_{\mathrm{obj}};\epsilon_{l}\right),$$

where smaller $\epsilon_{v}$ or $\epsilon_{l}$ means stronger privacy protection but potentially weaker legitimate recovery, whereas larger $P_{\mathrm{tx}}$ improves received SNR and thus favors semantic decoding. The detailed realizations of $M_{v}\left(\cdot \right)$ and $M_{l}\left(\cdot \right)$ are deferred to Section 3, where the DP mechanisms and their theoretical guarantees are given. The joint optimization of $P_{\mathrm{tx}}$, $\epsilon_{v}$, and $\epsilon_{l}$ is deferred to Section 4. Moreover, the notation $\left(\tilde{V},{\tilde{D}}_{\mathrm{obj}}\right)$ is used interchangeably with $\left(V_{\mathrm{tx}},D_{\mathrm{tx}}\right)$ when discussing the concrete mechanisms in Section 3.

## 3. Differential-Privacy-Based Visual and Location Protection Mechanisms

### 3.1. Overall DP Protection Architecture

Section 2 introduced the transmitter-side semantic messages V and $D_{\mathrm{obj}}$, the air–ground channel model, and the legitimate/eavesdropper observation boundaries. This section specifies the concrete privacy mechanisms $M_{v}\left(\cdot \right)$ and $M_{l}\left(\cdot \right)$ that act on these messages before transmission, and provides the corresponding theoretical guarantees. The optimization of the mechanism parameters $\left(P_{\mathrm{tx}},\epsilon_{v},\epsilon_{l}\right)$ is deferred to Section 4.

**Figure 2:**
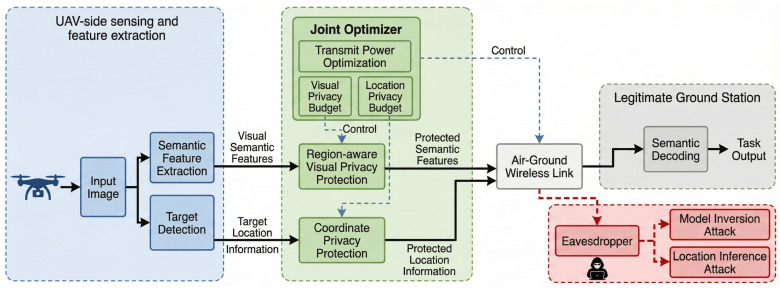
Overall framework of the dual privacy-enhancement scheme within the end-to-end UAV semantic communication chain.

Unlike Figure 1, which emphasizes the system scene and the privacy risks, Figure 2 presents the implementation-level mechanism architecture. In Figure 2, the left block performs UAV-side sensing and semantic feature extraction, the middle block contains the visual and location privacy mechanisms, and the right side shows the legitimate ground station and the eavesdropper after air–ground transmission. In this section, we focus on DP-based mechanisms for protecting visual semantic information and coordinate information.

### 3.2. Region-Aware Visual Differential Privacy Mechanism

The visual protection branch reduces attacker-side recoverability by injecting stronger perturbation into sensitive semantic cells while keeping weaker perturbation in non-critical regions [13,15]. For a detected object with box $B_{j}=\left(x_{j},y_{j},w_{j},h_{j}\right)$, the corner-coordinate form is

(8) $$B_{j}^{\mathrm{cor}}=\left(x_{1,j},y_{1,j},x_{2,j},y_{2,j}\right),$$

where $x_{1,j}=x_{j}-w_{j}/2$, $y_{1,j}=y_{j}-h_{j}/2$, $x_{2,j}=x_{j}+w_{j}/2$, and $y_{2,j}=y_{j}+h_{j}/2$. Let $s_{x}=W^{\prime }/W_{in}$ and $s_{y}=H^{\prime }/H_{in}$ denote the image-to-feature scaling factors. The projected box on the feature grid is

(9) $${\hat{B}}_{j}=\left(\left\lfloor s_{x}x_{1,j}\right\rfloor ,\left\lfloor s_{y}y_{1,j}\right\rfloor ,\left\lceil s_{x}x_{2,j}\right\rceil ,\left\lceil s_{y}y_{2,j}\right\rceil \right).$$

Let $\Omega_{\mathrm{obj},j}=G\left({\hat{B}}_{j}\right)$ denote the grid cells covered by the projected box and let $\Omega_{\mathrm{obj}}={⋃}_{j=1}^{M}\Omega_{\mathrm{obj},j}$ denote the overall object region.

To suppress contextual leakage in addition to object-interior leakage, the mechanism constructs an expanded context ring around sensitive categories. Let $J_{\mathrm{sen}}$ be the index set of sensitive objects. The context region is defined by

(10) $$\Omega_{\mathrm{ctx}}=\left(\underset{j\in J_{\mathrm{sen}}}{⋃}G\left({\hat{B}}_{j}^{\mathrm{ctx}}\right)\right)\setminus \Omega_{\mathrm{obj}},$$

and the remaining cells form the background region $\Omega_{\mathrm{bg}}$.

Instead of using a single global privacy coefficient, the method constructs a class-conditioned cell-wise privacy map from the visual budget $\epsilon_{v}$. Let $\epsilon_{u,v}^{\mathrm{map}}$ denote the privacy budget assigned to cell (u,v). The mechanism uses

(11) $$\epsilon_{u,v}^{\mathrm{map}}=\left\{\begin{array}{cc} \epsilon_{\mathrm{cls}}\left(l_{j};\epsilon_{v}\right), & \left(u,v\right)\in \Omega_{\mathrm{obj},j},\\ \kappa_{\mathrm{ctx}}\epsilon_{v}, & \left(u,v\right)\in \Omega_{\mathrm{ctx}},\\ \kappa_{\mathrm{bg}}\epsilon_{v}, & \left(u,v\right)\in \Omega_{\mathrm{bg}}, \end{array}\right.$$

where $\epsilon_{\mathrm{cls}}\left(l_{j};\epsilon_{v}\right)$ assigns smaller budgets to more sensitive categories, and $\kappa_{\mathrm{ctx}}=0.52$ and $\kappa_{\mathrm{bg}}=1.12$ control the protection strengths of the context ring and background, respectively. The class-conditioned budget function is defined as

(12) $$\epsilon_{\mathrm{cls}}\left(l_{j};\epsilon_{v}\right)=\max\;\left(\kappa_{l_{j}}\cdot \epsilon_{v}\cdot s_{l_{j}},\;\epsilon_{\min}\right),$$

where $\kappa_{l_{j}}$ is a per-class sensitivity coefficient, $s_{l_{j}}=0.3$ for the most privacy-sensitive categories (pedestrian and person) and $s_{l_{j}}=1.0$ otherwise, and $\epsilon_{\min}=0.08$ is a minimum noise floor that prevents degenerate noise. Table 2 lists the $\kappa_{l_{j}}$ values used in the experiments; smaller effective $\epsilon_{\mathrm{cls}}/\epsilon_{v}$ corresponds to stronger noise on that category.

To bound local sensitivity, each feature vector $v_{u,v}\in R^{C}$ is clipped before noise injection:

(13) $${\bar{v}}_{u,v}=\frac{v_{u,v}}{\max\left(1,\frac{∥v_{u,v}{∥}_{2}}{C_{\mathrm{clip}}}\right)}.$$

The clipped vector satisfies $∥{\bar{v}}_{u,v}{∥}_{2}\leq C_{\mathrm{clip}}$, and the resulting $l_{2}$ sensitivity is bounded by $\Delta f=2C_{\mathrm{clip}}$. The protected semantic tensor is then released as

(14) $${\tilde{V}}_{u,v}={\bar{v}}_{u,v}+n_{u,v},\;n_{u,v}∼N\left(0,\sigma_{u,v}^{2}I\right),$$

with

(15) $$\sigma_{u,v}=\frac{2C_{\mathrm{clip}}\sqrt{2\ln(1.25/\delta )}}{\epsilon_{u,v}^{\mathrm{map}}}.$$

**Proposition** **1.** *For each spatial cell (u,v), if the local feature vector is clipped to satisfy $∥{\bar{v}}_{u,v}{∥}_{2}\leq C_{\mathrm{clip}}$ and released with Gaussian noise variance $\sigma_{u,v}^{2}$ defined above, then the release ${\tilde{V}}_{u,v}$ satisfies $\left(\epsilon_{u,v}^{\mathrm{map}},\delta \right)$-DP with respect to neighbouring feature maps differing only at that cell. Consequently, the released protected semantic representation admits a cell-wise DP guarantee, and any subsequent channel transmission, legitimate decoding, or attacker-side processing preserves this guarantee by post-processing.*

**Proof.**  After clipping, the $l_{2}$ sensitivity of the local release at cell (u,v) is bounded by $\Delta f=2C_{\mathrm{clip}}$. The Gaussian mechanism therefore guarantees $\left(\epsilon_{u,v}^{\mathrm{map}},\delta \right)$-DP when the noise standard deviation is chosen as $\sigma_{u,v}=2C_{\mathrm{clip}}\sqrt{2\ln(1.25/\delta )}/\epsilon_{u,v}^{\mathrm{map}}$. Since the protection is applied cell-wise and all later operations only process the released tensor, the subsequent channel model, semantic decoder, and attacker model are all post-processing steps and cannot weaken the DP guarantee. □

Algorithm 1 summarizes the corresponding perturbation pipeline, and outputs the protected semantic tensor $\tilde{V}$, which serves as the visual semantic message transmitted over the air–ground link. At the algorithmic level, Algorithm 1 is a mechanism-level routine: under a given visual privacy budget $\epsilon_{v}$, it generates the protected semantic tensor $\tilde{V}$ and does not itself optimize transmit power or privacy-budget allocation.
**Algorithm 1** Region-Aware Visual Differential Privacy Mechanism**Require:** semantic feature tensor $V\in R^{C\times H^{\prime }\times W^{\prime }}$, detected object set $O={\left\{O_{j}\right\}}_{j=1}^{M}$ with boxes $B_{j}=\left(x_{j},y_{j},w_{j},h_{j}\right)$ and labels $l_{j}$, sensitive-object index set $J_{\mathrm{sen}}$ induced by sensitive categories, global privacy budget $\epsilon_{v}$, class-conditioned budget generator $\epsilon_{\mathrm{cls}}\left(l_{j};\epsilon_{v}\right)$, context and background coefficients $\kappa_{\mathrm{ctx}},\kappa_{\mathrm{bg}}$, clipping norm $C_{\mathrm{clip}}$, failure probability δ**Ensure:** protected semantic tensor $\tilde{V}$  1:Initialize the cell-wise budget map by $\epsilon_{u,v}^{\mathrm{map}}\leftarrow \kappa_{\mathrm{bg}}\epsilon_{v}$ for all feature-grid cells  2:Initialize $\Omega_{\mathrm{obj}}\leftarrow ⌀$ and $\Omega_{\mathrm{ctx}}\leftarrow ⌀$  3:**for** each detected object $O_{j}$ **do**  4:    Convert $B_{j}$ from center form to corner form $B_{j}^{\mathrm{cor}}$  5:    Project $B_{j}^{\mathrm{cor}}$ onto the feature grid to obtain ${\hat{B}}_{j}$  6:    Compute the object region $\Omega_{\mathrm{obj},j}\leftarrow G\left({\hat{B}}_{j}\right)$ and update $\Omega_{\mathrm{obj}}\leftarrow \Omega_{\mathrm{obj}}\cup \Omega_{\mathrm{obj},j}$  7:**end for**  8:**for** each detected object $O_{j}$ **do**  9:    **for** each cell $\left(u,v\right)\in \Omega_{\mathrm{obj},j}$ **do**10:        $\epsilon_{u,v}^{\mathrm{map}}\leftarrow \min\left\{\epsilon_{u,v}^{\mathrm{map}},\epsilon_{\mathrm{cls}}\left(l_{j};\epsilon_{v}\right)\right\}$11:    **end for**12:**end for**13:**for** each $j\in J_{\mathrm{sen}}$ **do**14:    Apply class-dependent expansion to the sensitive box and map it to the feature grid to obtain ${\hat{B}}_{j}^{\mathrm{ctx}}$15:    Compute the context candidate $\Omega_{\mathrm{ctx},j}^{\mathrm{cand}}\leftarrow G\left({\hat{B}}_{j}^{\mathrm{ctx}}\right)\setminus \Omega_{\mathrm{obj}}$16:    Update $\Omega_{\mathrm{ctx}}\leftarrow \Omega_{\mathrm{ctx}}\cup \Omega_{\mathrm{ctx},j}^{\mathrm{cand}}$17:    **for** each cell $\left(u,v\right)\in \Omega_{\mathrm{ctx},j}^{\mathrm{cand}}$ **do**18:        $\epsilon_{u,v}^{\mathrm{map}}\leftarrow \min\left\{\epsilon_{u,v}^{\mathrm{map}},\kappa_{\mathrm{ctx}}\epsilon_{v}\right\}$19:    **end for**20:**end for**21:Set the background region as $\Omega_{\mathrm{bg}}\leftarrow \left\{1,\ldots ,H^{\prime }\right\}\times \left\{1,\ldots ,W^{\prime }\right\}\setminus \left(\Omega_{\mathrm{obj}}\cup \Omega_{\mathrm{ctx}}\right)$22:**for** each feature-grid cell (u,v) **do**23:    Clip the local feature vector by     ${\bar{v}}_{u,v}\leftarrow v_{u,v}/\max\;\left(1,{∥v_{u,v}∥}_{2}/C_{\mathrm{clip}}\right)$24:    Compute the Gaussian scale     $\sigma_{u,v}\leftarrow 2C_{\mathrm{clip}}\sqrt{2\ln(1.25/\delta )}/\epsilon_{u,v}^{\mathrm{map}}$25:    Sample $n_{u,v}∼N\left(0,\sigma_{u,v}^{2}I\right)$ and set     ${\tilde{V}}_{u,v}\leftarrow {\bar{v}}_{u,v}+n_{u,v}$26:**end for**27:**return** $\tilde{V}$

### 3.3. Scenario-Adaptive Location Privacy Protection

The location privacy branch protects the object-center descriptors transmitted together with the visual semantic tensor. Unlike the visual branch in Section 3.2, the location branch adopts one of two DP mechanisms according to scenario requirements: a grid-based discrete release for coarse spatial awareness and a planar Laplace perturbation for continuous-coordinate protection.

#### 3.3.1. Randomized Differential Privacy for Discrete Grid-Based Location Information

The first location branch is designed for scenarios that prefer coarse spatial awareness while still requiring a formal DP guarantee. Let $G_{N}=\left\{\left(a,b\right):a,b\in \left\{0,\ldots ,N-1\right\}\right\}$ denote the set of all cells in an N×N grid. For each normalized object center $c_{j}=\left(x_{j},y_{j}\right)\in {\left[0,1\right]}^{2}$, the transmitter first identifies its reference grid cell

(16) $$g_{j}=\left(a_{j},b_{j}\right)=\left(\left\lfloor Nx_{j}\right\rfloor ,\left\lfloor Ny_{j}\right\rfloor \right).$$

Unlike deterministic grid quantization, the present branch does not release $g_{j}$ directly. Instead, it applies an exponential-mechanism-style randomized selection over all candidate cells in $G_{N}$ [24].

For any candidate grid cell $g=\left(a,b\right)\in G_{N}$, define the score function

(17) $$q\left(g,g_{j}\right)=-d_{1}\left(g,g_{j}\right),\;d_{1}\left(g,g_{j}\right)=|a-a_{j}|+|b-b_{j}|,$$

where $d_{1}\left(\cdot ,\cdot \right)$ is the Manhattan distance in grid steps. The protected output cell ${\tilde{g}}_{j}$ is sampled according to

(18) $$\mathrm{Pr}\left({\tilde{g}}_{j}=g|g_{j}\right)=\frac{\exp\;\left(\frac{\epsilon_{l}}{2}q\left(g,g_{j}\right)\right)}{\sum_{g^{\prime }\in G_{N}}\exp\;\left(\frac{\epsilon_{l}}{2}q\left(g^{\prime },g_{j}\right)\right)}=\frac{\exp\;\left(-\frac{\epsilon_{l}}{2}d_{1}\left(g,g_{j}\right)\right)}{\sum_{g^{\prime }\in G_{N}}\exp\;\left(-\frac{\epsilon_{l}}{2}d_{1}\left(g^{\prime },g_{j}\right)\right)}.$$

After sampling ${\tilde{g}}_{j}=\left({\tilde{a}}_{j},{\tilde{b}}_{j}\right)$, the released location descriptor is the grid center

(19) $${\tilde{c}}_{j}=\left(\frac{{\tilde{a}}_{j}+0.5}{N},\frac{{\tilde{b}}_{j}+0.5}{N}\right),\;{\mathrm{id}}_{j}={\tilde{b}}_{j}N+{\tilde{a}}_{j}.$$

Hence the branch still outputs grid centers with identifiers, but now under a randomized DP mechanism rather than deterministic nearest-cell assignment. This structured randomized release is also consistent with recent trajectory-data publishing studies, where adaptive grid constructions are combined with weighted differential privacy to balance privacy guarantees and spatial utility in sequential trajectory release [18].

**Proposition** **2.** *The above DP grid-based location mechanism satisfies discrete geo-indistinguishability on the grid. Specifically, for any two reference cells g and $g^{\prime }$ in $G_{N}$ and any output cell $z\in G_{N}$, the mechanism obeys*

(20) $$\frac{\mathrm{Pr}(\tilde{g}=z|g)}{\mathrm{Pr}(\tilde{g}=z|g^{\prime })}\leq \exp\;\left(\epsilon_{l}d_{1}\left(g,g^{\prime }\right)\right).$$

*Therefore, the branch satisfies formal location-DP on the discrete grid.*

**Proof.**  By construction,

(21) $$\mathrm{Pr}\left(\tilde{g}=z|g\right)=\frac{\exp\;\left(-\frac{\epsilon_{l}}{2}d_{1}\left(z,g\right)\right)}{Z(g)},\;Z\left(g\right)=\sum_{y\in G_{N}}\exp\;\left(-\frac{\epsilon_{l}}{2}d_{1}\left(y,g\right)\right).$$

Using the triangle inequality of $d_{1}\left(\cdot ,\cdot \right)$, we have $d_{1}\left(z,g\right)\geq d_{1}\left(z,g^{\prime }\right)-d_{1}\left(g,g^{\prime }\right)$, so

(22) $$\exp\;\left(-\frac{\epsilon_{l}}{2}d_{1}\left(z,g\right)\right)\leq \exp\;\left(\frac{\epsilon_{l}}{2}d_{1}\left(g,g^{\prime }\right)\right)\exp\;\left(-\frac{\epsilon_{l}}{2}d_{1}\left(z,g^{\prime }\right)\right).$$

Likewise, for any candidate *y*, $d_{1}\left(y,g^{\prime }\right)\leq d_{1}\left(y,g\right)+d_{1}\left(g,g^{\prime }\right)$, which implies

(23) $$Z\left(g^{\prime }\right)\geq \exp\;\left(-\frac{\epsilon_{l}}{2}d_{1}\left(g,g^{\prime }\right)\right)Z\left(g\right).$$

Combining the two inequalities yields

(24) $$\frac{\mathrm{Pr}(\tilde{g}=z|g)}{\mathrm{Pr}(\tilde{g}=z|g^{\prime })}\leq \exp\;\left(\epsilon_{l}d_{1}\left(g,g^{\prime }\right)\right),$$

which proves the stated discrete geo-indistinguishability guarantee. □

#### 3.3.2. Laplace-Based Differential Privacy for Continuous Coordinates Location Information

The second location branch is designed for scenarios that require continuous perturbation rather than discrete grid outputs. For each object center $c_{j}=\left(x_{j},y_{j}\right)$, the perturbation vector $\Delta c_{j}=\left(\Delta x_{j},\Delta y_{j}\right)$ follows the two-dimensional planar Laplace density, as commonly adopted in geo-indistinguishability and recent semantic-aware location privacy studies [25,26]

(25) $$f_{\Delta c}\left(\Delta x,\Delta y\right)=\frac{\epsilon_{l}^{2}}{2\pi }\exp\;\left(-\epsilon_{l}\sqrt{\Delta x^{2}+\Delta y^{2}}\right).$$

Equivalently, in polar form, $\theta_{j}∼\mathrm{Uniform}\left(0,2\pi \right)$ and the radial marginal is

(26) $$p_{r}\left(r\right)=\epsilon_{l}^{2}re^{-\epsilon_{l}r},\;r\geq 0.$$

A practical implementation may sample perturbations in physical units and map them back to normalized coordinates by a fixed scaling constant. This constant can be absorbed into the effective privacy parameter and therefore does not change the mechanism class. The released coordinate is then written as

(27) $${\tilde{x}}_{j}=\mathrm{clip}\left(x_{j}+\Delta x_{j},0,1\right),\;{\tilde{y}}_{j}=\mathrm{clip}\left(y_{j}+\Delta y_{j},0,1\right),$$

with ${\tilde{c}}_{j}=\left({\tilde{x}}_{j},{\tilde{y}}_{j}\right)$.

**Proposition** **3.** *The planar Laplace mechanism above satisfies $\epsilon_{l}$-geo-indistinguishability. Specifically, for any two true coordinates c and $c^{\prime }$ and any released coordinate $\tilde{c}$ in the continuous plane,*

(28) $$\frac{f(\tilde{c}|c)}{f(\tilde{c}|c^{\prime })}\leq \exp\;\left(\epsilon_{l}{∥c-c^{\prime }∥}_{2}\right).$$

**Proof.**  The conditional density of the planar Laplace mechanism is proportional to $\exp(-\epsilon_{l}∥\tilde{c}-c{∥}_{2})$. Hence

(29) $$\frac{f(\tilde{c}|c)}{f(\tilde{c}|c^{\prime })}=\exp\;\left(\epsilon_{l}\left(∥\tilde{c}-c^{\prime }{∥}_{2}-{∥\tilde{c}-c∥}_{2}\right)\right).$$

By the triangle inequality, $∥\tilde{c}-c^{\prime }{∥}_{2}-∥\tilde{c}{-c∥}_{2}\leq {∥c-c^{\prime }∥}_{2}$, which gives

(30) $$\frac{f(\tilde{c}|c)}{f(\tilde{c}|c^{\prime })}\leq \exp\;\left(\epsilon_{l}{∥c-c^{\prime }∥}_{2}\right).$$

Therefore the mechanism satisfies $\epsilon_{l}$-geo-indistinguishability. □

#### 3.3.3. Scenario-Adaptive Mechanism Selection and Implementation Algorithm

The location branch selector $B_{\mathrm{loc}}^{\left(s\right)}$ determines which location mechanism is used in each mission scenario. In the current design, the surveillance and resource-limited scenarios employ the DP grid-based location mechanism so as to preserve coarse region awareness with bounded message complexity, whereas the precision-oriented scenario employs the planar Laplace branch so as to retain continuous spatial perturbation capability.

Algorithm 2 summarizes this scenario-adaptive location protection procedure. The algorithm outputs either a randomized grid center with identifier or a continuously perturbed coordinate descriptor. Together with the protected visual tensor $\tilde{V}$, these outputs form the protected semantic messages defined in Section 2. The joint optimization of the operating budgets that drive Algorithms 1 and 2, together with the transmit power, is presented in Section 4.
**Algorithm 2** Scenario-Adaptive DP Location Protection**Require:** normalized object centers ${\left\{c_{j}=\left(x_{j},y_{j}\right)\right\}}_{j=1}^{M}$, branch selector $B_{\mathrm{loc}}^{\left(s\right)}$, location privacy budget $\epsilon_{l}$, grid size *N*, candidate grid set $G_{N}$, coordinate scaling factor $s_{c}$**Ensure:** protected location descriptors, namely randomized grid centers with identifiers or perturbed normalized coordinates  1:**if** $B_{\mathrm{loc}}^{\left(s\right)}$ selects the DP grid-based location mechanism **then**  2:    **for** each object center $c_{j}=\left(x_{j},y_{j}\right)$ **do**  3:        Compute the reference grid cell $g_{j}\leftarrow \left(\left\lfloor Nx_{j}\right\rfloor ,\left\lfloor Ny_{j}\right\rfloor \right)$  4:        **for** each candidate cell $g\in G_{N}$ **do**  5:           Compute the score $q\left(g,g_{j}\right)\leftarrow -d_{1}\left(g,g_{j}\right)$  6:           Assign the sampling weight $w\left(g\right)\leftarrow \exp\;\left(\frac{\epsilon_{l}}{2}q\left(g,g_{j}\right)\right)$  7:        **end for**  8:        Sample the protected cell ${\tilde{g}}_{j}$ from $G_{N}$ with probability proportional to w(g)  9:        Convert ${\tilde{g}}_{j}=\left({\tilde{a}}_{j},{\tilde{b}}_{j}\right)$ to the released grid center     ${\tilde{c}}_{j}\leftarrow \left(\left({\tilde{a}}_{j}+0.5\right)/N,\left({\tilde{b}}_{j}+0.5\right)/N\right)$10:        Set the randomized grid identifier ${\mathrm{id}}_{j}\leftarrow {\tilde{b}}_{j}N+{\tilde{a}}_{j}$11:    **end for**12:    **return** ${\left\{\left({\tilde{c}}_{j},{\mathrm{id}}_{j}\right)\right\}}_{j=1}^{M}$13:**else**14:    **for** each object center $c_{j}=\left(x_{j},y_{j}\right)$ **do**15:        Sample $\theta_{j}∼\mathrm{Uniform}\left(0,2\pi \right)$16:        Sample $r_{j}$ from the planar-Laplace radial marginal     $p_{r}\left(r\right)=\epsilon_{l}^{2}re^{-\epsilon_{l}r},\;r\geq 0$17:        Set the protected normalized coordinate by     ${\tilde{x}}_{j}\leftarrow \mathrm{clip}\;\left(x_{j}+r_{j}\cos\theta_{j}/s_{c},0,1\right)$     ${\tilde{y}}_{j}\leftarrow \mathrm{clip}\;\left(y_{j}+r_{j}\sin\theta_{j}/s_{c},0,1\right)$18:        Set ${\tilde{c}}_{j}\leftarrow \left({\tilde{x}}_{j},{\tilde{y}}_{j}\right)$19:    **end for**20:    **return** ${\left\{{\tilde{c}}_{j}\right\}}_{j=1}^{M}$21:**end if**

## 4. Joint Transmission Power and Privacy Budget Optimization

Given the system model in Section 2 and the concrete DP protection mechanisms in Section 3, this section formulates the optimization problem over the transmit power $P_{\mathrm{tx}}$ and the mechanism parameters $\epsilon_{v}$ and $\epsilon_{l}$. In other words, the visual and location privacy mechanisms are already fixed by Section 3; the task of the present section is to choose their operating budgets jointly with the transmit power under scenario-dependent utility and privacy constraints.

### 4.1. Scenario-Dependent Joint Optimization Problem

The optimization problem is built from three quantities. Let $A_{\mathrm{sem}}\left(\epsilon_{v},\gamma \right)$ denote the legitimate-side semantic task quality after protection and transmission. The dependence on $P_{\mathrm{tx}}$ is represented through the induced SNR γ, so $P_{\mathrm{tx}}$ remains a direct optimization variable through the channel model and power constraints rather than appearing twice in the semantic-quality term. The semantic task quality function takes the log-utility form

(31) $$A_{\mathrm{sem}}\left(\epsilon_{v},\gamma \right)=\alpha \log_{2}\;\left(1+\frac{hP_{\mathrm{tx}}}{\sigma_{\mathrm{ch}}^{2}}\right)-\frac{\beta }{\epsilon_{v}},$$

where α=0.5 weights the SNR contribution and β=0.8 penalises the utility loss from stronger DP noise; both are monotone and concave in their respective arguments, consistent with diminishing returns of SNR and privacy-budget allocations.

Let $E_{\mathrm{loc}}\left(\epsilon_{l}\right)$ denote the location error induced by the location-protection mechanism. The location utility takes the log form

(32) $$U_{\mathrm{loc}}\left(\epsilon_{l}\right)=\gamma \log_{2}\left(1+\epsilon_{l}\right),\;\gamma =0.8,$$

which is monotone increasing in $\epsilon_{l}$ (looser protection → smaller location error → higher utility).

Since semantic quality and location error are measured on different scales, the joint utility is constructed from normalized terms. Let $A_{\mathrm{ref}}$ denote a reference upper semantic-quality level under an admissible high-utility operating regime. The normalized semantic utility and normalized location cost are defined as

(33) $${\bar{A}}_{\mathrm{sem}}=\mathrm{clip}\;\left(\frac{A_{\mathrm{sem}}\left(\epsilon_{v},\gamma \right)-A_{\min}}{A_{\mathrm{ref}}-A_{\min}},0,1\right),$$

(34) $${\bar{E}}_{\mathrm{loc}}=\mathrm{clip}\;\left(\frac{E_{\mathrm{loc}}\left(\epsilon_{l}\right)}{E_{\max}},0,1\right),$$

where $A_{\min}$ is the minimum acceptable semantic-quality threshold and $E_{\max}$ is the maximum tolerable location error. The corresponding normalized location utility is

(35) $$U_{\mathrm{loc}}\left(\epsilon_{l}\right)=1-{\bar{E}}_{\mathrm{loc}}\left(\epsilon_{l}\right).$$

This normalization places the semantic and location terms on a common dimensionless scale before they are combined.

Let *s* denote the mission scenario. Each scenario is associated with the policy tuple

(36) $$\Theta^{\left(s\right)}=\left\{\lambda_{1}^{\left(s\right)},\lambda_{2}^{\left(s\right)},P_{\max}^{\left(s\right)},\epsilon_{\mathrm{total}}^{\left(s\right)},A_{\min}^{\left(s\right)},E_{\max}^{\left(s\right)},B_{\mathrm{loc}}^{\left(s\right)}\right\},$$

where $\lambda_{1}^{\left(s\right)}$ and $\lambda_{2}^{\left(s\right)}$ weight semantic and location utility, $P_{\max}^{\left(s\right)}$ and $\epsilon_{\mathrm{total}}^{\left(s\right)}$ define the resource budgets, and $B_{\mathrm{loc}}^{\left(s\right)}$ denotes the location-protection branch selected by the scenario policy. The corresponding optimal values of the optimization variables are written as

(37) $$\pi^{*(s)}=\left\{P_{\mathrm{tx}}^{*(s)},\epsilon_{v}^{*(s)},\epsilon_{l}^{*(s)}\right\},$$

and the scenario-dependent utility maximization problem is formulated as

(38) $$\max_{P_{\mathrm{tx}},\epsilon_{v},\epsilon_{l}}\;U^{\left(s\right)}=\lambda_{1}^{\left(s\right)}{\bar{A}}_{\mathrm{sem}}\left(\epsilon_{v},\gamma \right)+\lambda_{2}^{\left(s\right)}U_{\mathrm{loc}}\left(\epsilon_{l}\right),$$

subject to

(39) $$0\leq P_{\mathrm{tx}}\leq P_{\max}^{\left(s\right)},$$

(40) $$\epsilon_{v}+\epsilon_{l}\leq \epsilon_{\mathrm{total}}^{\left(s\right)},$$

(41) $$A_{\mathrm{sem}}\left(\epsilon_{v},\gamma \right)\geq A_{\min}^{\left(s\right)},$$

(42) $$E_{\mathrm{loc}}\left(\epsilon_{l}\right)\leq E_{\max}^{\left(s\right)}.$$

The surveillance, precision-oriented, and resource-limited scenarios differ in utility weights, budget constraints, minimum utility requirements, and branch selection.

The mapping from $\left(P_{\mathrm{tx}},\epsilon_{v},\epsilon_{l}\right)$ to semantic utility, location utility, and privacy risk is nonlinear and scenario-dependent. Recent adaptive semantic transmission and resource-constrained edge-inference studies likewise emphasize joint adaptation of communication resources, model partition, and task utility [4,23]. Section 3 instantiates the visual and location protection mechanisms, while this section formalizes the resulting joint optimization problem and solves it with a BCD-based iterative algorithm. The solver implements the above formulation and does not imply a closed-form solution of the original non-convex problem.

### 4.2. BCD-Based Joint Optimization Algorithm

This subsection numerically addresses the scenario-dependent control problem over $\left(P_{\mathrm{tx}},\epsilon_{v},\epsilon_{l}\right)$ in the spirit of recent adaptive semantic transmission and edge-inference optimization studies [4,23]. Because $P_{\mathrm{tx}}$ affects the received SNR and the privacy budgets jointly affect semantic distortion and location utility, the resulting optimization is non-convex. A BCD-based approximate iterative solver is therefore adopted rather than a closed-form exact solution.

At iteration *t*, subproblem A updates the dual privacy budgets under fixed transmit power:

(43) $$\left({\hat{\epsilon }}_{v}^{\left(t+1\right)},{\hat{\epsilon }}_{l}^{\left(t+1\right)}\right)=\arg\max_{\epsilon_{v},\epsilon_{l}}U^{\left(s\right)}\;\left(P_{\mathrm{tx}}^{\left(t\right)},\epsilon_{v},\epsilon_{l}\right),$$

subject to

(44) $$\epsilon_{v}+\epsilon_{l}\leq \epsilon_{\mathrm{total}}^{\left(s\right)},\;A_{\mathrm{sem}}\left(\epsilon_{v},\gamma \right)\geq A_{\min}^{\left(s\right)},\;E_{\mathrm{loc}}\left(\epsilon_{l}\right)\leq E_{\max}^{\left(s\right)}.$$

Subproblem A is solved through a one-dimensional search over $\epsilon_{v}$, with $\epsilon_{l}=\epsilon_{\mathrm{total}}^{\left(s\right)}-\epsilon_{v}$, which is sufficient because the total privacy budget couples the two variables directly.

Subproblem B then updates the transmit power under the refreshed privacy budgets:

(45) $${\hat{P}}_{\mathrm{tx}}^{\left(t+1\right)}=\arg\max_{0<P_{\mathrm{tx}}\leq P_{\max}^{\left(s\right)}}U^{\left(s\right)}\;\left(P_{\mathrm{tx}},\epsilon_{v}^{\left(t+1\right)},\epsilon_{l}^{\left(t+1\right)}\right),$$

which is consistent with the transmit-power budget in Section 2. Numerically, this step is solved through a one-dimensional search over an effective feasible interval obtained after accounting for fixed circuit consumption and privacy-processing overhead. This search interval is an implementation-level realization of the same power budget rather than a different optimization constraint.

To improve numerical stability, the solver applies damping after both subproblems:

(46) $$\epsilon_{v}^{\left(t+1\right)}=\eta {\hat{\epsilon }}_{v}^{\left(t+1\right)}+\left(1-\eta \right)\epsilon_{v}^{\left(t\right)},\;\epsilon_{l}^{\left(t+1\right)}=\epsilon_{\mathrm{total}}^{\left(s\right)}-\epsilon_{v}^{\left(t+1\right)},$$

(47) $$P_{\mathrm{tx}}^{\left(t+1\right)}=\eta {\hat{P}}_{\mathrm{tx}}^{\left(t+1\right)}+\left(1-\eta \right)P_{\mathrm{tx}}^{\left(t\right)},$$

where the damping factor is set to η=0.55. Let $U^{\left(t\right)}$ denote the utility value at iteration *t*. The stopping rule is

(48) $$\left|U^{\left(t+1\right)}-U^{\left(t\right)}\right|<10^{-4},$$

where the maximum iteration number is set to 50. 

#### Convergence Analysis

The BCD solver is guaranteed to produce a non-decreasing utility sequence ${\left\{U^{\left(t\right)}\right\}}_{t\geq 1}$ under the following argument. Sub-problem A performs an exact one-dimensional search over the compact feasible interval $\left[\epsilon_{v,\min},\epsilon_{\mathrm{total}}-\epsilon_{l,\min}\right]$, so the update ${\hat{\epsilon }}_{v}^{\left(t+1\right)}$ cannot decrease *U* relative to the previous $\left(\epsilon_{v}^{\left(t\right)},\epsilon_{l}^{\left(t\right)},P_{\mathrm{tx}}^{\left(t\right)}\right)$. Sub-problem B similarly maximises *U* over $\left[0,P_{\max}\right]$. Damping with factor η∈(0,1) preserves this monotonicity in expectation and prevents oscillation between near-optimal points. Because the feasible set is compact and *U* is bounded, the sequence $\left\{U^{\left(t\right)}\right\}$ converges. In practice, convergence is reached within 6–8 iterations in all tested scenarios (see Section 5.9), with the utility gap $|U^{\left(t+1\right)}-U^{\left(t\right)}|$ dropping below the tolerance $10^{-4}$ well before the maximum of 50 iterations.

Algorithm 3 summarizes the complete alternating update procedure as the upper-level optimization routine, while Algorithms 1 and 2 remain the fixed mechanism-level protection modules evaluated at each candidate operating point. The output of this module is the optimization variables $\left(P_{\mathrm{tx}},\epsilon_{v},\epsilon_{l}\right)$ used by the visual and location protection branches for the corresponding transmission block. Therefore, Algorithm 3 provides the optimized variables, Algorithm 1 realizes visual DP protection under $\epsilon_{v}$, and Algorithm 2 realizes location protection under $\epsilon_{l}$ and $B_{\mathrm{loc}}^{\left(s\right)}$.
**Algorithm 3** BCD-Based Joint Power-Privacy Optimization over the Instantiated Protection Mechanisms**Require:** scenario policy tuple $\Theta^{\left(s\right)}$, channel gain *h*, noise variance $\sigma_{\mathrm{ch}}^{2}$, damping factor η=0.55, minimum iteration count $T_{\min}=6$, maximum iteration number $T_{\max}=50$, stopping tolerance $\tau =10^{-4}$**Ensure:** updated optimization variables $\left(P_{\mathrm{tx}}^{\left(s\right)},\epsilon_{v}^{\left(s\right)},\epsilon_{l}^{\left(s\right)}\right)$  1:Initialize $\left(P_{\mathrm{tx}}^{\left(0\right)},\epsilon_{v}^{\left(0\right)},\epsilon_{l}^{\left(0\right)}\right)$ from $\Theta^{\left(s\right)}$ and the channel realization  2:Compute $U^{\left(0\right)}\leftarrow U^{\left(s\right)}\left(P_{\mathrm{tx}}^{\left(0\right)},\epsilon_{v}^{\left(0\right)},\epsilon_{l}^{\left(0\right)}\right)$  3:**for** $t=0,1,\ldots ,T_{\max}-1$**do**  4:    $\gamma^{\left(t\right)}\leftarrow P_{\mathrm{tx}}^{\left(t\right)}h/\sigma_{\mathrm{ch}}^{2}$  5:    Solve Subproblem A by one-dimensional search over $\epsilon_{v}\in \left[0,\epsilon_{\mathrm{total}}^{\left(s\right)}\right]$     with $\epsilon_{l}=\epsilon_{\mathrm{total}}^{\left(s\right)}-\epsilon_{v}$ and constraints     $A_{\mathrm{sem}}\left(\epsilon_{v},\gamma^{\left(t\right)}\right)\geq A_{\min}^{\left(s\right)}$ and $E_{\mathrm{loc}}\left(\epsilon_{l}\right)\leq E_{\max}^{\left(s\right)}$  6:    Obtain $\left({\hat{\epsilon }}_{v}^{\left(t+1\right)},{\hat{\epsilon }}_{l}^{\left(t+1\right)}\right)$  7:    $\epsilon_{v}^{\left(t+1\right)}\leftarrow \eta {\hat{\epsilon }}_{v}^{\left(t+1\right)}+\left(1-\eta \right)\epsilon_{v}^{\left(t\right)}$  8:    $\epsilon_{l}^{\left(t+1\right)}\leftarrow \epsilon_{\mathrm{total}}^{\left(s\right)}-\epsilon_{v}^{\left(t+1\right)}$  9:    Solve Subproblem B by one-dimensional search over $0<P_{\mathrm{tx}}\leq P_{\max}^{\left(s\right)}$     and tighten the implementation-level search interval by fixed circuit consumption     and privacy-processing overhead10:    Obtain ${\hat{P}}_{\mathrm{tx}}^{\left(t+1\right)}$11:    $P_{\mathrm{tx}}^{\left(t+1\right)}\leftarrow \eta {\hat{P}}_{\mathrm{tx}}^{\left(t+1\right)}+\left(1-\eta \right)P_{\mathrm{tx}}^{\left(t\right)}$12:    $U^{\left(t+1\right)}\leftarrow U^{\left(s\right)}\left(P_{\mathrm{tx}}^{\left(t+1\right)},\epsilon_{v}^{\left(t+1\right)},\epsilon_{l}^{\left(t+1\right)}\right)$13:    **if** $t+1\geq T_{\min}$ and $\left|U^{\left(t+1\right)}-U^{\left(t\right)}\right|<\tau$ **then**14:        **break**15:    **end if**16:**end for****Handoff:** feed $\epsilon_{v}^{\left(t+1\right)}$ to Algorithm 1, feed $\epsilon_{l}^{\left(t+1\right)}$ together with $B_{\mathrm{loc}}^{\left(s\right)}$ to Algorithm 2, and use $P_{\mathrm{tx}}^{\left(t+1\right)}$ in the air–ground transmission stage17:**return**$\left(P_{\mathrm{tx}}^{\left(t+1\right)},\epsilon_{v}^{\left(t+1\right)},\epsilon_{l}^{\left(t+1\right)}\right)$

## 5. Simulation Results and Analysis

### 5.1. Dataset and Parameter Settings Across Different Scenarios

The experiments use the VisDrone dataset and are conducted on the official validation split [27], which is the standard protocol used in the VisDrone detection and tracking benchmark. To ensure a consistent statistical basis across all reported comparisons, each scenario-level or privacy–utility summary is aggregated over 500 validation samples drawn from this split. For the attacker-side reconstruction evaluation, the results are reported as mean ± 95% confidence interval computed over 100 attack cases per DP mode (20 images × 5 seeds), providing statistically reliable evidence of the privacy protection effectiveness across diverse image content.

Three scenario policies are studied. The surveillance scenario uses $\epsilon_{\mathrm{total}}=5.0$ and $P_{\max}=2.0$ W, emphasizes balanced sensing utility under a high-risk environment, and adopts the DP grid-based location mechanism. The precision-oriented scenario uses a tighter privacy budget $\epsilon_{\mathrm{total}}=1.5$ together with a larger power limit $P_{\max}=5.0$ W and a planar Laplace branch, which reflects tasks requiring higher semantic fidelity and link quality. The resource-limited scenario sets $\epsilon_{\mathrm{total}}=3.0$ and $P_{\max}=0.5$ W, and adopts the DP grid-based location mechanism to emphasize conservative resource usage.

### 5.2. Baseline Schemes Design

Three complementary experimental protocols are considered. The first protocol evaluates the proposed region-aware pipeline under the surveillance, precision-oriented, and resource-limited policies, and is used for the cross-scenario comparison. The second protocol fixes the location privacy budget at $\epsilon_{l}=1.0$ and sweeps the visual privacy budget over $\epsilon_{v}\in \left\{0.3,0.5,0.8,1.0,1.5,2.0,3.0\right\}$ to compare uniform differential privacy with region-aware differential privacy. The third protocol fixes $\epsilon_{v}=1.0$ and $\epsilon_{l}=1.0$ in the surveillance scenario and compares no protection, uniform differential privacy, and region-aware differential privacy.

The adopted baselines serve different purposes. No protection provides an upper-bound reference for legitimate semantic utility while exposing the privacy risk of transmitting unprotected features. Uniform differential privacy serves as a non-selective privacy baseline and tests whether privacy can be improved without exploiting semantic-region sensitivity. Region-aware differential privacy is then compared against these references to isolate the benefit of spatially selective perturbation. All compared methods share the same feature extractor, the same semantic decoder, the same channel model, the same task setting, and the same attack-side evaluation protocol. Therefore, the reported differences can be attributed to the protection mechanism or the scenario policy rather than to inconsistent budgets, links, or datasets.

### 5.3. Evaluation Metrics

Overall utility measures the final system-level privacy–utility trade-off achieved by the joint optimization. Semantic category accuracy quantifies semantic usability at the legitimate receiver after privacy protection and air–ground transmission. Sensitive-region SSIM and sensitive-feature cosine similarity quantify attacker-side visual recoverability at the structural and feature levels, respectively. Location error measures the cost introduced by the location-protection mechanism, and received SNR characterizes the communication condition supporting semantic decoding. Attack-side PSNR and SSIM are also reported to quantify reconstruction suppression under different visual privacy mechanisms [13].

### 5.4. Hyperparameter Selection

The key hyperparameters are set as follows. The grid size N=16 for the discrete location DP branch is chosen to provide a spatial resolution of approximately 1/16 of the normalized coordinate range, which balances grid granularity with the sensitivity of the exponential-mechanism normalization constant. Finer grids (larger *N*) provide higher utility at the cost of a smaller effective privacy amplification from randomization. The coordinate scaling factor that maps continuous normalized coordinates to the grid is absorbed into the effective privacy parameter $\epsilon_{l}$ and does not change the mechanism class. For the visual DP branch, the context-ring scale $\kappa_{\mathrm{ctx}}=0.52$ and background scale $\kappa_{\mathrm{bg}}=1.12$ are set so that the context ring receives approximately half the noise of the most sensitive object cells ($\kappa_{\mathrm{ctx}}=0.52<1$), and the background receives slightly weaker noise than the global budget ($\kappa_{\mathrm{bg}}=1.12>1$ yields $\epsilon_{\mathrm{bg}}^{\mathrm{map}}=1.12\epsilon_{v}$, corresponding to $\sigma_{\mathrm{bg}}=\sigma \left(\epsilon_{v}\right)/1.12\approx 89\%$ of the global-budget noise standard deviation); these values were selected to maintain task utility while suppressing contextual leakage around sensitive regions and ensuring that background noise does not fall to zero. The clipping norm $C_{\mathrm{clip}}=1.0$ is set to bound the $l_{2}$ sensitivity of each feature vector, and $\delta =10^{-5}$ follows the standard choice for Gaussian mechanism analysis in machine-learning settings.

### 5.5. Implementation Details and Statistical Protocol

All experiments are conducted in a unified Python 3.10/PyTorch 2.1.0 simulation environment and can run on either CPU or CUDA devices. The same semantic feature extraction, privacy protection, air–ground transmission, and semantic decoding pipeline is used across all compared methods, and the compared settings differ only in the privacy mechanism or the scenario policy. This task configuration is consistent with recent visual semantic communication studies that evaluate task-level semantic output quality under shared transmission and task heads rather than pixel-level fidelity [4,6,10]. This unified configuration ensures fair comparison across baselines and across scenario policies.

For optimization, the BCD-based solver uses a stopping tolerance of $10^{-4}$, a maximum of 50 iterations, a minimum of 6 iterations before the stopping criterion is activated, and a damping factor of η=0.55. For visual privacy, three protection modes are considered, namely no protection, uniform differential privacy, and region-aware differential privacy, and the Gaussian mechanism uses $\delta =10^{-5}$. On the location side, the protection branch is selected by the scenario policy and takes the form of either the DP grid-based location mechanism or the planar Laplace branch. The scenario-dependent privacy budgets and transmit-power budgets follow the scenario definitions stated above.

The fixed-budget evaluation reported in Section 5.8 uses $\epsilon_{v}=1.0$ and a gradient-based reconstruction reference. At $\epsilon_{v}=1.0$, the region-aware DP applies noise with σ≈32 to the most sensitive cells (pedestrians), under which the trained inversion network produces near-random reconstructions across all DP modes and cannot discriminate between mechanism designs; the gradient-based attacker is therefore used in that evaluation as a controlled ablation indicator. Section Adversarial Evaluation: Gradient-Based and Trained Inversion Attackers uses the trained inversion network at $\epsilon_{v}\in \left\{10,20\right\}$, a regime in which the attacker achieves partial reconstruction under no-protection and can meaningfully distinguish the selective protection behavior of region-aware DP from uniform DP.

### 5.6. Privacy–Utility Trade-Off Under Different Visual Privacy Budgets

**Figure 3:**
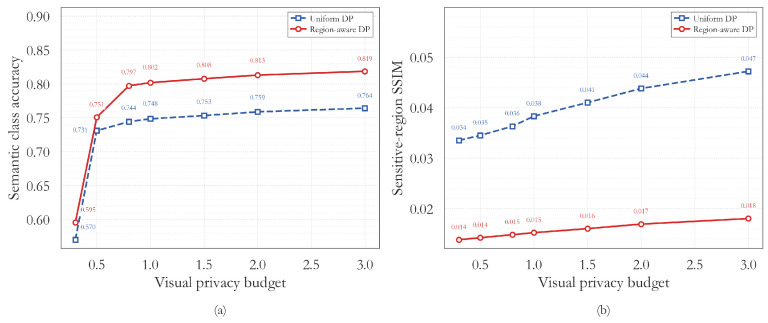
Privacy–utility trade-off under different visual privacy budgets: (**a**) semantic class accuracy and (**b**) sensitive-region SSIM.

Figure 3 shows the privacy–utility frontier under a fixed location privacy budget $\epsilon_{l}=1.0$ and a visual privacy sweep over $\epsilon_{v}\in \left\{0.3,0.5,0.8,1.0,1.5,2.0,3.0\right\}$. Both uniform DP and region-aware DP improve semantic category accuracy as the visual privacy budget becomes looser. However, the region-aware mechanism remains consistently better positioned on the frontier.

The numerical results further clarify this gap. At $\epsilon_{v}=1.0$, region-aware DP achieves a semantic category accuracy of 0.802, compared with 0.748 under uniform DP, corresponding to a 7.2% relative improvement, while the sensitive-region SSIM is reduced from 0.038 to 0.015, corresponding to a 60.5% relative reduction. At $\epsilon_{v}=3.0$, region-aware DP still preserves a higher semantic category accuracy of 0.819 versus 0.764, corresponding to a 7.2% relative improvement, and meanwhile keeps the sensitive-region SSIM much lower at 0.018 versus 0.047, corresponding to a 61.7% relative reduction. The same trend is observed in sensitive-feature similarity, indicating that the proposed mechanism preserves more task-relevant semantics while suppressing attacker-side recovery more effectively under the same budget sweep.

### 5.7. Cross-Scenario Performance Under Heterogeneous Mission Priorities

**Figure 4:**
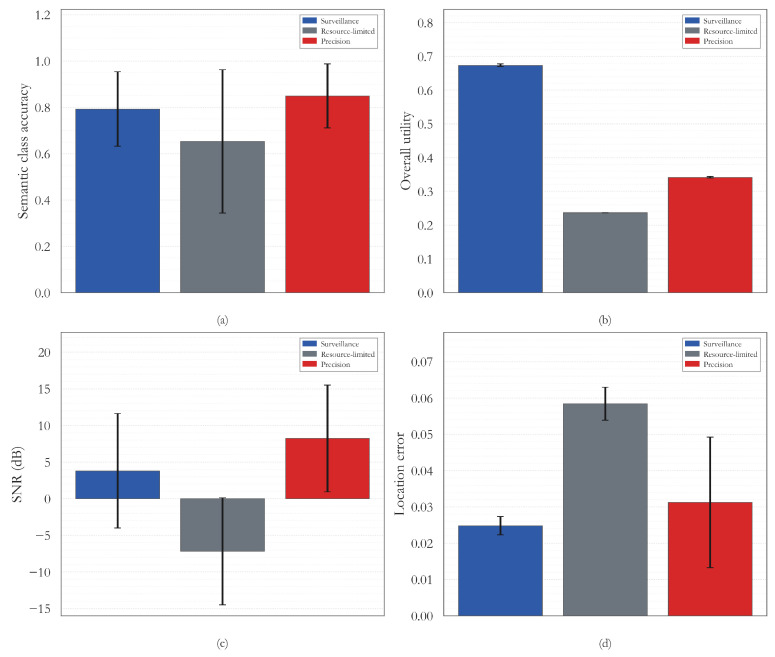
Performance comparison across different task scenarios: (**a**) semantic class accuracy, (**b**) overall utility, (**c**) received SNR, and (**d**) location error.

Figure 4 compares cross-scenario behavior under heterogeneous mission priorities. The same region-aware pipeline is evaluated under the surveillance, precision-oriented, and resource-limited policies. The surveillance scenario reaches the highest overall utility, with a mean value of 0.6733, while the precision-oriented and resource-limited scenarios obtain 0.3418 and 0.2365, respectively. This result shows that the proposed framework converges to different operating points according to scenario requirements rather than applying a fixed privacy-budget or transmit-power setting to all tasks.

The scenario-level means further explain these differences. The precision-oriented setting allocates a larger average transmit power of 0.2596 W and achieves the highest mean received SNR of 8.216 dB, which is consistent with its emphasis on semantic fidelity. The resource-limited setting reduces the average transmit power to 0.0094 W and correspondingly yields the lowest mean SNR of −7.184 dB, reflecting strict resource control. Meanwhile, the surveillance scenario keeps the lowest mean location error of 0.0248, compared with 0.0312 in the precision-oriented case and 0.0584 in the resource-limited case. These results confirm that the proposed joint optimization framework coordinates privacy budgets, communication quality, and location distortion according to mission priority rather than optimizing a single metric in isolation.

### 5.8. Fixed-Budget DP Ablation and Mechanism Interpretation

**Figure 5:**
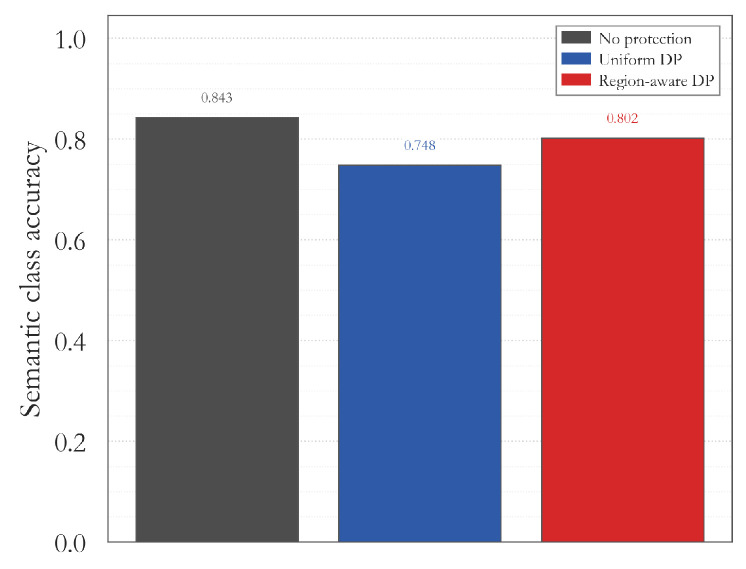
Semantic category accuracy under different visual protection mechanisms (no protection, uniform DP, region-aware DP) at fixed $\epsilon_{v}=1.0$, $\epsilon_{l}=1.0$ in the surveillance scenario. Higher accuracy indicates better preservation of legitimate-side task semantics.

**Figure 6:**
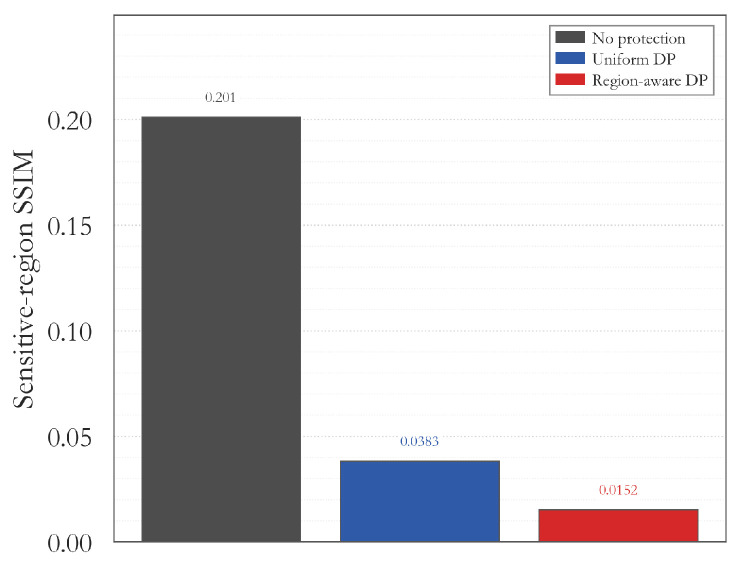
Attacker-side SSIM of sensitive semantic regions under different visual protection mechanisms at fixed $\epsilon_{v}=1.0$, $\epsilon_{l}=1.0$. Lower sensitive-region SSIM indicates stronger suppression of attacker visual recovery within object-centric areas.

**Figure 7:**
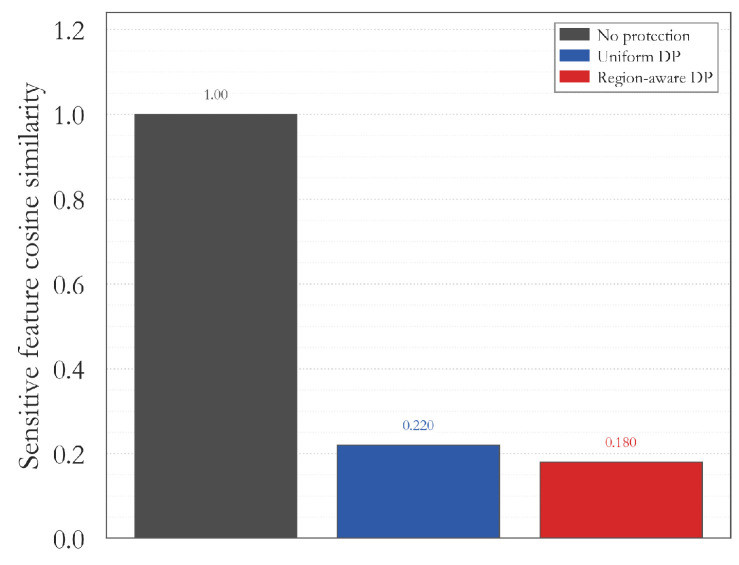
Cosine similarity of sensitive semantic features between the intercepted protected representation and the original features, under different visual protection mechanisms at fixed $\epsilon_{v}=1.0$, $\epsilon_{l}=1.0$. Lower similarity indicates that the attacker’s recovered representation is semantically further from the original, reflecting stronger privacy protection at the feature level.

Figure 5, Figure 6 and Figure 7 report the fixed-budget comparison under the surveillance scenario with $\epsilon_{v}=1.0$ and $\epsilon_{l}=1.0$, where no protection, uniform DP, and region-aware DP are evaluated under the same setting. Because the privacy budgets and scenario policy are fixed while the visual protection strategy changes, this comparison directly reveals the contribution of the visual privacy mechanism.

The numerical results show a clear three-way trade-off. Without protection, the semantic category accuracy is 0.843, but the sensitive-region SSIM reaches 0.201 and the sensitive-feature similarity remains 1.0, indicating almost complete recoverability on the attacker side. Uniform DP suppresses the sensitive-region SSIM to 0.0383 and reduces the sensitive-feature similarity to 0.22, but it also lowers the semantic category accuracy to 0.748. Region-aware DP improves the semantic category accuracy to 0.802 while further reducing the sensitive-region SSIM to 0.0152 and the sensitive-feature similarity to 0.18. Relative to uniform DP, this means a 7.2% improvement in semantic category accuracy, a 60.3% reduction in sensitive-region SSIM, and an 18.2% reduction in sensitive-feature similarity. At the whole-image attack level, the attack SSIM under region-aware DP is 0.01687, which is comparable to the 0.01708 of uniform DP, but is achieved with noticeably better legitimate-task performance.

These comparisons show that region-aware perturbation is the key visual-protection mechanism of the proposed framework. Relative to uniform DP, it preserves more legitimate-task semantics while further suppressing sensitive-region leakage, with simultaneous gains in task accuracy and reductions in both structural and feature-level recoverability. Relative to no protection, it sharply reduces attacker-side recoverability while maintaining a substantially stronger task-side response than uniform perturbation under the same fixed-budget setting.

#### Adversarial Evaluation: Gradient-Based and Trained Inversion Attackers

This section evaluates attacker-side recoverability under two complementary threat models introduced in Section 2.5. The gradient-based optimization attacker serves as a lower-bound baseline: it iteratively minimizes the L1 distance between the feature map of a candidate image and the intercepted protected feature, starting from a random initialization, without any training data (300 optimization steps per image). The trained inversion network is a strictly stronger adversary that pre-trains a lightweight convolutional inverter $I:R^{C\times H^{\prime }\times W^{\prime }}\;\to R^{3\times H\times W}$ on 450 training images drawn from the same distribution, then applies it at inference time to reconstruct the original image from intercepted semantic features (no channel noise, direct feature interception). Two metrics are reported: Attack SSIM ↓, the full-image structural similarity of the attacker’s reconstruction relative to the original; and Sensitive-region PSNR ↓, the peak signal-to-noise ratio computed exclusively on pixels inside ground-truth sensitive bounding boxes. All results cover 100 independent runs per condition (20 test images × 5 random seeds) and are reported as mean ± 95% CI via the *t*-distribution (Table 3).

Table 3 presents results for both attacker formulations. The gradient-based optimizer achieves Attack SSIM =0.0939±0.0022 under no protection, confirming that even training-free iterative optimization can extract limited structural information from unprotected features. The trained inversion network, with access to 450 in-distribution training images, achieves Attack SSIM =0.5483±0.0071 under the same condition—nearly 6× higher—establishing it as the dominant reconstruction threat. Both DP mechanisms substantially degrade both attackers. For the trained inverter at ε=10, Uniform DP reduces Attack SSIM by 74% (0.5483→0.1410; 95% CIs non-overlapping); for the gradient attacker, region-aware DP achieves a 77% reduction (0.0939→0.0217). At the sensitive-region level, the region-aware mechanism provides additional selective protection: at ε=10, sensitive-region PSNR reaches 9.53±0.14 dB (trained inverter) and 9.83±0.09 dB (gradient attacker), both substantially lower than Uniform DP (12.73±0.20 dB and 10.66±0.11 dB respectively; 95% CIs non-overlapping). At the full-image level, region-aware DP yields a higher Attack SSIM than Uniform DP for the trained inverter at both ε values (0.1835 vs. 0.1652 at ε=20; 0.1571 vs. 0.1410 at ε=10). This reflects the inherent selectivity trade-off: by concentrating noise on sensitive regions while applying weaker perturbation to background areas ($\kappa_{\mathrm{bg}}=1.12$), region-aware DP intentionally allows the attacker to recover background structure more accurately in exchange for substantially stronger protection of privacy-sensitive content. The protection advantage of region-aware DP is therefore selective rather than global, targeting the semantic content that carries the greatest privacy risk.

### 5.9. Convergence Behavior and Optimization Overhead

**Figure 8:**
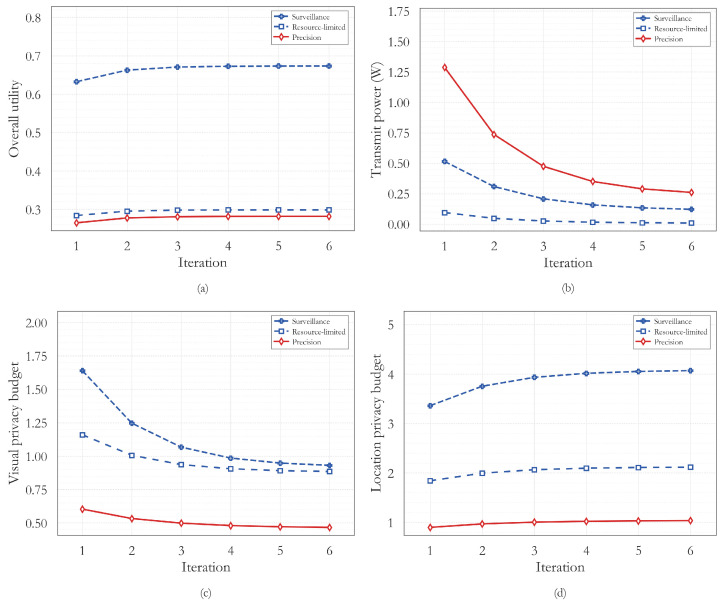
Convergence curves of the jointly optimized variables: (**a**) overall utility, (**b**) transmit power, (**c**) visual privacy budget, and (**d**) location privacy budget.

Figure 8 reports the BCD-based joint optimization dynamics. Because the optimizer uses a minimum of 6 iterations, a convergence tolerance of $10^{-4}$, and an upper bound of 50 iterations, the recorded histories can be directly interpreted as optimization-overhead evidence. In the precision-oriented scenario, the mean utility increases from 0.2649 at iteration 1 to 0.2817 at iteration 6. In the resource-limited scenario, the mean utility rises from 0.2839 to 0.2986 over the same interval. In the surveillance scenario, the mean utility grows from 0.6325 at iteration 1 to 0.6733 at iteration 6.

Most cases already stabilize within six to seven iterations. Only a very small subset of surveillance samples continues to an eighth iteration, where the mean utility reaches 0.6801 for the remaining four hard cases. These observations indicate that the proposed joint optimization reaches stable operating points with a short iteration horizon and moderate iteration-level overhead.

### 5.10. Qualitative Semantic Output Visualization, Privacy Visualization, and Limitations

**Figure 9:**
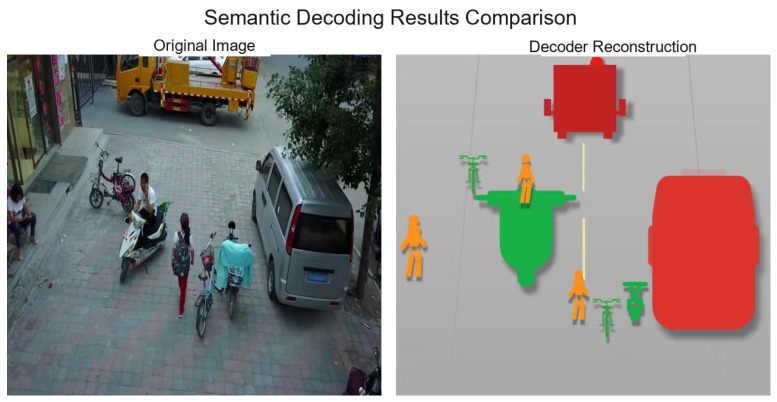
Comparison between the original image and a task-level semantic output visualization rendered from the decoder mask. Different colors denote different detected categories (orange: pedestrian; green: non-motor vehicle; red: motor vehicle).

Figure 9 compares the original image with a task-level semantic output visualization rendered from the decoder mask at the legitimate receiver. The right panel is not a pixel-level reconstructed image; it is a visualization generated from the decoder’s semantic output to highlight object categories and coarse spatial layout. It shows that the protected features preserve the relative positions and semantic distribution of major objects such as vehicles, pedestrians, bicycles, and coarse scene structure after air–ground transmission. Because this visualization is derived from task-level semantic masks rather than photorealistic rendering, fine-grained textures, colors, and detailed visual appearances are intentionally absent. This observation is consistent with the design goal of semantic communication, namely preserving task-oriented semantics rather than directly recognizable visual details.

**Figure 10:**
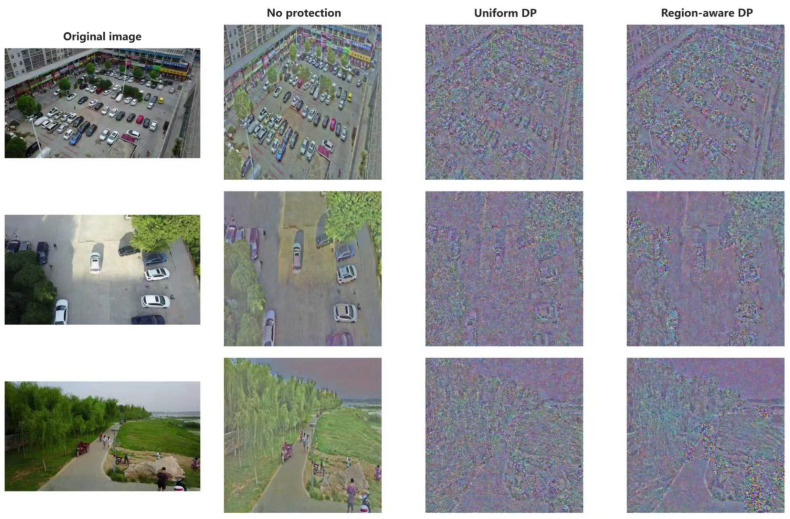
Qualitative comparison of DP-perturbed feature reconstructions under different visual privacy protection mechanisms, rendered via the gradient-based reconstruction reference.

Figure 10 provides qualitative comparisons of multiple scenes under no protection, uniform differential privacy, and region-aware differential privacy. Under no protection, vehicle contours, parking layouts, and road textures remain relatively clear, indicating that the attacker may still recover sensitive visual content with identifying value. Uniform differential privacy significantly perturbs the whole image and weakens the visibility of sensitive content, but it also destroys a considerable amount of non-sensitive background structure. By contrast, region-aware differential privacy introduces stronger perturbations over sensitive target regions such as vehicles while preserving more global scene outlines such as roads, vegetation, and the overall layout. This qualitative evidence visually supports the quantitative observations in Figure 5, Figure 6 and Figure 7.

### 5.11. Computational Complexity

Table 4 reports the measured runtime and peak memory overhead of the three core modules.

The DP protection modules add negligible per-frame overhead relative to the semantic encoding and decoding pipeline. The BCD solver converges within 6–8 iterations in all reported scenarios (Figure 8), with each iteration performing a one-dimensional search over a compact interval for each sub-problem.

### 5.12. Simulation-to-Deployment Gap

The experiments are conducted in a controlled simulation environment; we explicitly characterise the key gaps relative to practical UAV deployment. (i) Detection accuracy: the simulation assumes a YOLO detector with performance matched to the VisDrone benchmark; in practice, detection accuracy may vary with altitude, lighting, and motion blur, which would affect the sensitive-region mask used by the visual DP mechanism. (ii) Channel realism: the Rayleigh block-fading model captures small-scale multipath fading but does not account for interference, blockage, or Doppler spread under high UAV mobility; extensions to time-varying and interference-limited channel models remain future directions. (iii) Onboard computation: the complexity measurements in Section 5.11 indicate that the DP and BCD modules add negligible latency, but end-to-end deployment on constrained UAV hardware requires hardware-level profiling beyond the simulation environment. Practical UAV deployment and hardware validation are listed as priority future directions.

Taken together, the quantitative and qualitative results indicate that the proposed framework achieves a favorable privacy–utility trade-off and differentiated operating points across task scenarios. Across trade-off evaluation, cross-scenario adaptation, fixed-budget mechanism comparison, convergence behavior, and representative visual cases, the method consistently preserves more task-relevant semantics while reducing attacker-side recoverability. Robustness analyses over channel quality, DP noise scaling, scene density, and attack preprocessing conditions would further broaden the evaluation scope.

## 6. Conclusions

This paper presents a collaborative UAV semantic communication framework for jointly protecting visual semantics and location information under communication-resource constraints. The framework couples dual-privacy modeling with joint optimization of transmit power, the visual privacy budget, and the location privacy budget, thereby establishing a unified privacy–utility trade-off for the legitimate receiver and attacker-side recoverability. The experimental evaluation compares the proposed framework against no-protection and uniform DP baselines: relative to uniform DP, region-aware DP improves semantic category accuracy while further suppressing sensitive-region recoverability under the same fixed privacy budget, and relative to no protection, it sharply reduces attacker-side recoverability while maintaining substantially stronger legitimate-task performance than uniform DP. The results also show that the proposed method reaches stable operating points within a small number of iterations and supports differentiated protection behavior under surveillance, precision-oriented, and resource-limited scenarios. 

## Figures and Tables

**Table 1:** Principal symbols used throughout the paper.

Symbol	Meaning
I	Input UAV image
E(·)	Transmitter-side semantic encoder and detector
O	Detected object set
$B_{j}$	Bounding box of object *j*
$l_{j}$	Category label of object *j*
V	Visual semantic feature tensor
G(·)	Descriptor organization operator
$D_{\mathrm{obj}}$	Raw target-related descriptor set
$V_{\mathrm{tx}}$	Visual semantic tensor placed on the air–ground link
$D_{\mathrm{tx}}$	Target-related descriptor set placed on the air–ground link
$M_{v}\left(\cdot \right)$	Visual privacy mechanism
$M_{l}\left(\cdot \right)$	Location privacy mechanism
$\tilde{V}$	Protected visual semantic feature tensor
${\tilde{D}}_{\mathrm{obj}}$	Protected target-related descriptor set
${\tilde{c}}_{j}$	Protected coordinate descriptor of object *j*
C(·)	Air–ground transmission operator
*h*	Effective air–ground channel gain
$\sigma_{\mathrm{ch}}^{2}$	Channel noise variance
γ	Received SNR of the air–ground link
$\hat{V}$	Received semantic feature tensor at the legitimate receiver
${\hat{D}}_{\mathrm{obj}}$	Received descriptor set at the legitimate receiver
$P_{\det}$	Detector-derived prior cues reconstructed from received descriptors
$D_{\mathrm{sem}}\left(\cdot \right)$	Legitimate-side semantic decoder
$\hat{M}$	Legitimate-side task-oriented semantic output
A(·)	Eavesdropper-side inference or attack operator
${\hat{I}}_{\mathrm{adv}}$	Attacker-side recovered visual content
$\epsilon_{v}$	Visual privacy budget
$\epsilon_{l}$	Location privacy budget
$P_{\mathrm{tx}}$	UAV transmit power
$B_{\mathrm{loc}}^{\left(s\right)}$	Location-protection branch selected by mission scenario *s*

**Table 2:** Per-class sensitivity coefficient $\kappa_{l_{j}}$ in the class-conditioned privacy-budget map.

Category	$\kappa_{l_{j}}$	Effective $\epsilon_{\mathrm{cls}}/\epsilon_{v}$
Pedestrian, Person	1.00	0.30
Bicycle	1.38	1.38
Motor, Tricycle, Awning-Tricycle	1.50–1.55	1.50–1.55
Truck	1.72	1.72
Car	1.82	1.82
Van	1.88	1.88
Bus	1.95	1.95
Context ring ($\kappa_{\mathrm{ctx}}$)	—	0.52
Background ($\kappa_{\mathrm{bg}}$)	—	1.12

**Table 3:** Dual-attacker evaluation (100 runs per condition; mean ± 95% CI, *t*-distribution). Attack SSIM ↓: full-image SSIM of the attacker’s reconstruction vs. the original. Sen.-region PSNR ↓: PSNR on sensitive-region pixels only. Lower values indicate stronger privacy protection.

DP Mode	ε	Attacker	Attack SSIM ↓	Sen.-Region PSNR (dB) ↓
No Protection	—	Trained Inverter	0.5483±0.0071	18.53±0.21
No Protection	—	Gradient Optim	0.0939±0.0022	12.20±0.16
Uniform DP	20	Trained Inverter	0.1652±0.0016	13.15±0.20
Uniform DP	20	Gradient Optim	0.0540±0.0014	11.23±0.12
Uniform DP	10	Trained Inverter	0.1410±0.0015	12.73±0.20
Uniform DP	10	Gradient Optim	0.0366±0.0009	10.66±0.11
Region-Aware DP	20	Trained Inverter	0.1835±0.0028	9.50±0.18
Region-Aware DP	20	Gradient Optim	0.0254±0.0003	10.04±0.10
Region-Aware DP	10	Trained Inverter	0.1571±0.0027	9.53±0.14
Region-Aware DP	10	Gradient Optim	0.0217±0.0003	9.83±0.09

**Table 4:** Measured runtime (mean ± std, ms) and peak memory (KB) of the three core modules, averaged over 200 repeated trials on a single CPU core.

Module	Time (ms)	Peak Mem (KB)
Region-aware DP (per frame)	1.41±0.24	504.7
Coordinate DP (per frame)	0.10±0.05	2.1
BCD optimization (total, ≤8 iters)	19.0±5.5	2.3
BCD optimization (per iteration)	2.38	—

## Data Availability

The raw data supporting the conclusions of this article will be made available by the authors on request.
